# Changing R&D models in research-based pharmaceutical companies

**DOI:** 10.1186/s12967-016-0838-4

**Published:** 2016-04-27

**Authors:** Alexander Schuhmacher, Oliver Gassmann, Markus Hinder

**Affiliations:** School of Applied Chemistry, Reutlingen University, Alteburgstrasse 150, 72762 Reutlingen, Germany; Institute of Technology Management, University of St. Gallen, Dufourstrasse 40a, 9000 St. Gallen, Switzerland; Novartis Institutes for BioMedical Research, Postfach, Forum 1, 4002 Basel, Switzerland

## Abstract

New drugs serving unmet medical needs are one of the key value drivers of research-based pharmaceutical companies. The efficiency of research and development (R&D), defined as the successful approval and launch of new medicines (output) in the rate of the monetary investments required for R&D (input), has declined since decades. We aimed to identify, analyze and describe the factors that impact the R&D efficiency. Based on publicly available information, we reviewed the R&D models of major research-based pharmaceutical companies and analyzed the key challenges and success factors of a sustainable R&D output. We calculated that the R&D efficiencies of major research-based pharmaceutical companies were in the range of USD 3.2–32.3 billion (2006–2014). As these numbers challenge the model of an innovation-driven pharmaceutical industry, we analyzed the concepts that companies are following to increase their R&D efficiencies: (A) Activities to reduce portfolio and project risk, (B) activities to reduce R&D costs, and (C) activities to increase the innovation potential. While category A comprises measures such as portfolio management and licensing, measures grouped in category B are outsourcing and risk-sharing in late-stage development. Companies made diverse steps to increase their innovation potential and open innovation, exemplified by open source, innovation centers, or crowdsourcing, plays a key role in doing so. In conclusion, research-based pharmaceutical companies need to be aware of the key factors, which impact the rate of innovation, R&D cost and probability of success. Depending on their company strategy and their R&D set-up they can opt for one of the following open innovators: knowledge creator, knowledge integrator or knowledge leverager.

## Background

The importance of research and development (R&D) for the pharmaceutical industry is evidenced by the cumulative R&D expenditure in this sector as a whole but also on the individual company level. The total worldwide R&D spend of pharmaceutical and biotechnology companies increased from USD 108 billion (2006) to USD 141 billion (2015) [1]. Amongst the world top 50 companies by total R&D investment in the fiscal year 2014/2015 were 16 pharmaceutical companies. Novartis (5), Roche (7), Johnson & Johnson (J&J, 8) and Pfizer (10) ranked in the top 10 of the leading R&D investing companies globally [2]. Accordingly, the pharmaceutical industry is a worldwide top investor in R&D today and it is predicted that it will keep its role as a leading R&D stakeholder in the future with an industry-wide forecasted total R&D spend of USD 160 billion by 2020 [1]. It is predicted that Novartis (10.5), Roche (9.1), Pfizer (7.5), Merck & Co. (7.1), J&J (6.7), Sanofi (6.1), AstraZeneca (5.6) and GlaxoSmithKline (GSK, 5.4) will still allocate more then USD 5 billion on R&D in 2020 [1].

The challenge related to the high R&D spend is the rising expectations of investors for a reasonable return of investment (ROI) provided by a high number of new molecular entities (NMEs) launched to the major pharmaceutical markets. Although exceptions exist, the industry as a whole did not live up to these expectations, as the total number of NMEs commercialized in past years did not match with the extraordinary high R&D costs. Measured by the number of NMEs approved by the US Food and Drug Administration (FDA), most of the top pharmaceutical companies did not launch enough new drugs in the past years to achieve the reported 2–3 NMEs/year/company which would be necessary to achieve the growth objectives based on product innovation [3–9]. In consequence, this misbalance put a question mark to the long-term sustainability of the industry’s R&D model and forced the big companies in the industry to search for other growth options and potential savings.

This article reviews the efficiency parameters of pharmaceutical R&D and the consequences of the low R&D input/output-ratio for the industry. Moreover, it illustrates and exemplifies the role of open innovation in the pharmaceutical sector. Last, it outlines why a change in the R&D model is required and which models pharmaceutical companies may follow to increase the productivity of their R&D organizations.^1^

## The risks of pharmaceutical R&D

The Center for Medicine Research International (CMR) reported in its Pharmaceutical R&D Factbook 2014 an average success rate of 4.9 % from first toxicity dose to market approval with between phase success rates of 66, 44, 26, 72 and 91 % from first toxicity dose to first human dose, first human dose to first patient dose, first patient dose to first pivotal dose, first pivotal dose to first submission and first submission to first launch, respectively [11]. Paul et al. [12] reported success rates of 51 % for discovery research, 69 % for preclinical development, 12.8 % for the clinical development phases and 91 % for the submission phase, resulting in an overall probability of technical and regulatory success (PTRS) for drug R&D of 4.1 % [12].

In general, the reasons of the reported high attrition rates are diverse and comprise [13]:

- lack of reliability of published data [14],
- biopharmaceutical issues including suboptimal PK [4],
- poorly predictive preclinical models in discovery research and preclinical testing [15],
- the concept of target-based drug discovery with the related advanced complexity of target selection, a competition for proprietary targets and the complex process of target validation, [15–19].
- complexity of clinical trials (to treat chronic diseases), together with increasing demands from regulatory authorities and payers, and
- the lack of know-how of smaller organizations resulting in a lower PTRS from Phase I to submission than large organizations [20].

The FDA approvals in 2012 have been reviewed in this context and lack of efficacy (56 %), safety issues (28 %), changing strategies (7 %), commercial reasons (5 %) and operational challenges (5 %) are the most probable reasons of failures that happened in phase II and phase III of clinical development [21]. These results were confirmed by a second analysis of 142 drug R&D projects of AstraZeneca [22]. Preclinical and phase I projects primarily failed for safety reasons and projects failing in phases II and III commonly lacked efficacy [22].

The higher target-specificity and reduced incidence of off-target effects of biologics, such as monoclonal antibodies, proteins, or peptides, for naturally occurring ligands suggests that these molecules might have higher chances of success than smaller molecules. Since 2004, several authors have compared the success rates for small molecules with those of biologics [4, 23–25]. All of them consistently found higher success rates for biologics. The likelihood of successful approval from phase I across all therapeutic areas and indications was in the 10 %-range. For biologics these phase transition rates from phase I to approval were higher [4, 23–25]. On the other hand, Hay et al. [25] provided data demonstrating that the status of being a lead indication or being an oncology project impacts the PTRS in the same way as the distinction between small molecules and biologics. Therefore and in our view, these findings need to be interpreted with caution. The sample sizes, especially for biologics, are relatively small and the unequal distribution of biologics across therapeutic areas and types of company (big pharma vs. biotech) might have biased the results. Additionally, some authors have described a wrong classification of biologics to be small molecules and vice versa which again biases the interpretation of available data.

## Interval durations for drug R&D

As of its direct link to opportunity cost and reduction of the patent life, overall R&D time and interval durations are of greatest interest as an R&D efficiency measure. According to Paul et al. [12], drug R&D (across all therapeutic areas) takes on average 14 years [12]. Discovery research lasts for 4.5 years, preclinical testing continues for 1 year, the three clinical development phases take 1.5, 2.5 and 2.5 years, respectively, and the phase from submission to launch requires another 18 months [12]. Interval durations for basic research and post-approval Phase IV trials need to be added to the overall R&D time to consider the entire pharmaceutical R&D process.

There are two additional findings when reviewing drug R&D timelines:

- Clinical development today takes more time than in the past. While the average clinical development time for drugs approved between 2005 and 2009 was 6.4 years [26], newer data from the 2014 CMR Factbook show that the composite median interval duration for ongoing development projects (2008–2012) is 9.1 years. The CMR data clearly show a trend toward increased interval durations in preclinical development (+17 %, 2004–2012) and in phase I of clinical development (+58 %, 2004–2012).
- The average time for the FDA review and approval has decreased significantly since the enactment of the Prescription Drug User Fee Act (PDUFA) [26, 27]. One aspect that may have contributed positively to faster review and approval timelines are the fast-track status or accelerated approvals for new drugs in indications with a high unmet medical need, such as in oncology.

The long overall time of pharmaceutical R&D impacts the total R&D costs, the risk of industry rivalry and the uncertainties of generic competition. First, investments in R&D projects were incurred many years ago and need to be capitalized till the date of ROI of the new drug. The capitalization of R&D costs results in an enormous increase in the overall R&D expenditures [12]. Second, as many pharmaceutical companies follow comparable strategies as to therapeutic areas, target diseases, biologic mechanisms and drug targets, the long R&D timelines increase the risk of competition, reduce the chance to be first-in-market, cut the market potential and the commercial success of a drug candidate. Third, the effective date of generic competition influences the ROI of a new drug, as any delay in drug development results in a reduction of the commercially usable patent term.

## Risks and time influence R&D costs negatively

The costs for pharmaceutical R&D increased in the past decades significantly. Munos [3] reported an annual inflation-adjusted increase of R&D costs of 8.6 % for the period of 1950–2009 [3]. Other studies support this view: while the costs per NME were published to be USD 250 million before the 1990s, the average out-of-the-pocket costs per NME have been calculated to be USD 403 million (2000s) and USD 873 million (2010), respectively [12, 28, 29]. The low success rates and the respective costs of failed drug projects are causal for the high out-of-the-pocket costs. In addition, the use of new technologies to reduce the timelines and to increase success rates in drug discovery, such as combinatorial chemistry, DNA sequencing, high-throughput-screening (HTS) or computational drug design, may have further increased R&D costs just as larger clinical trial sizes and better clinical infrastructure. Split to the phases of R&D, Paul et al. [12] reported that drug discovery and preclinical development account for 33 % of the total cost per NME (USD 281 million), clinical development (phase I to submission) represents 63 % (USD 548 million) and submission to launch costs 5 % (USD 44 million) of the overall expenditures per NME [12]. In view of the long time intervals, these out-of-the-pocket costs add together in extraordinary high capitalized costs of reported of USD 1.778 billion (2010) per NME [12, 19, 28].

Such cost calculations do not include all expenditures associated directly and indirectly with drug R&D. Costs for basic research, phase IV trials, regulatory approvals in non-US markets or product life-cycle management need to be added. Exemplified by data from the 2014 CMR Factbook that 25.7 % of all costs of R&D are dedicated to the international roll-out and line extensions, the actual costs per new drug are higher than the analyzed USD 1.778 billion. Potentially, the results provided by Harper [30] illustrate the real costs, as he analyzed the expenditures per NME launched by leading pharmaceutical companies to be USD 3-12 billion. And he investigated that the top pharmaceutical companies, defined as those that have launched more than four NMEs between 2002–2011, invested more than USD 5 billion per new drug.

## The actual challenge for the pharmaceutical industry

**Fig. 1:**
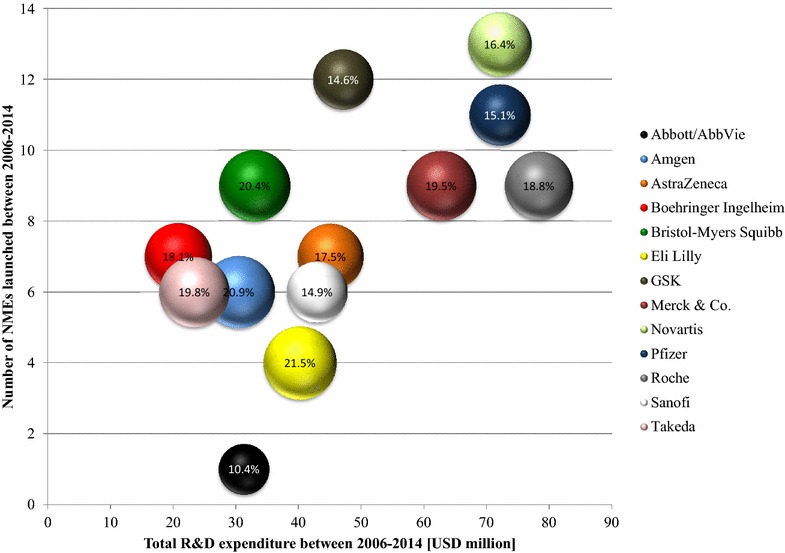
R&D efficiencies of research-based pharmaceutical companies (2006–2014). Total number of NMEs (new molecular entities) approved by the FDA contrasted to the total R&D expenditure per company between 2006–2014. *Bubble size* illustrates the R&D intensity (R&D expenditure/total sales) in a  %-rate. Merck & Co including Schering Plough (starting 2009), Pfizer including Wyeth (starting 2009), Roche including Genentech (starting 2010), Novartis including Alcon (starting 2010), Sanofi including Genzyme (starting 2011)

The actual challenge for the industry comes from putting the costs of pharmaceutical R&D in context to the output, namely the number of NMEs launched to the market. Scannell et al. [19] have analyzed the historical input/output-ratio of the pharmaceutical industry and concluded a bisection of the R&D efficiency every 9 years between 1950–2010 [19]. Although todays output of some research-based pharmaceutical companies is remarkable, such as the 13 NMEs launched by Novartis in the period of 2006–2014, contrasting the output figures per company to their total R&D expenditure of up to USD 80 billion (2006–2014) highlights the real challenge the companies are facing (see Fig. 1). Although such analyses comprise inherent inaccuracies, such as the input parameter does not match time-wise with the output correctly, it still shows the dilemma of the pharmaceutical industry.

In consequence and following the argumentation of Harper [30] the historical calculation of approximately USD 1 billion per approved drug needs to be corrected to an amount of more than USD 3 billion (see Table 1). In detail, Boehringer Ingelheim, Bristol-Myers Squibb, Takeda and GSK all spent USD 3–4 billion per NME, while Amgen, Novartis, AstraZeneca, Pfizer, Merck & Co., Sanofi and Roche each invested up to USD 8 billion per new drug approved by the FDA. And Eli Lilly and Abbott/AbbVie invested even more than USD 10 billion per NME (2006–2014)—an amount of money that at least for some pharmaceutical companies put a question mark on the sustainability of their R&D models.

**Table 1:** R&D efficiencies of multinational pharmaceutical companies (2006–2014)

	Total R&D expenditures (USD million) (2006–2014)	Number of FDA approved NMEs (2006–2014)	R&D efficiency (USD million/NME) (2006–2014)
Abbott/Abbvie	31,292	1	31,292
Eli Lilly	40,232	4	10,058
Roche	78,340	9	8704
Sanofi	42,948	6	7158
Merck & Co.	62,745	9	6972
Pfizer	72,125	11	6557
AstraZeneca	45,081	7	6440
Novartis	72,100	13	5546
Amgen	30,437	6	5073
GSK	47,109	12	3926
Takeda	23,361	6	3893
Bristol-Myers Squibb	33,006	9	3667
Boehringer Ingelheim	22,920	7	3274

Merck & Co including Schering Plough (starting 2009), Pfizer including Wyeth (starting 2009), Roche including Genentech (starting 2010), Novartis including Alcon (starting 2010), Sanofi including Genzyme (starting 2011)

Source: Annual company reports, http://www.fda.gov/downloads/Drugs/DevelopmentApprovalProcess/HowDrugsareDevelopedandApproved/DrugandBiologicApprovalReports/UCM081805.pdf

The reasons that have been discussed previously in the context of the high attrition rates and the interval durations also apply here. Further causes may have affected the R&D efficiency negatively, such as:

- an inadequate number of projects in early R&D phases [12],
- technically more complex research for new drug targets and subsequent preclinical and clinical studies [19],
- a higher burden for approval and reimbursement of NMEs in view of the already approved drugs,
- a lower risk tolerance of both regulators and society [19],
- the high number of mergers & acquisitions (M&As) [31–33, 35],
- the decreasing number of research-based pharmaceutical companies taking the financial risk of drug R&D [34] and
- a negative effect of licensing, co-development, or joint ventures on the clinical development and approval durations [35].

## Producing blockbusters could help

Even though the reasons for the low R&D efficiency are known, a sector-wide general concept to solve this problem does not exist so far. Quite simply, the low R&D efficiency could be compensated by an increase in the financial value per NME launched. Thus, the 4 % of successful projects that result in new commercialized drugs have to provide enough revenue to justify the investment of the 96 % failed compounds and to provide enough profit for the investors. Consequently, commercializing more blockbuster drugs would compensate the low output and the increasing costs of pharmaceutical R&D. This rather traditional concept of the sector has resulted in an overall industry portfolio of 48 blockbuster drugs marketed in 2014 with some drugs such as Humira (USD 11.0 billion, 2013), Enbrel (USD 8.75 billion, 2013) or Advair (USD 8.3 billion, 2013) providing extraordinary high annual sales of more than USD 5 billion. It is however expected that average peak sales per NME will not achieve blockbuster dimensions, reflecting the increased challenges of offering benefits over already existing treatments [15]. In the mature markets of Europe and the US, new products face stronger competition, need to be developed for better profiled patients populations, and are launched to smaller market segments which in turn reduces the market potential of the new drugs. At once, drugs face high cost pressure from the public and payers which affects the commercial potential negatively. For example, the public already discusses the justification for the prize of USD 84,000 per treatment regimen with Gilead’s blockbuster drug Solvadis for the treatment of Hepatitis C (http://www.wsj.com/articles/no-justification-for-solvadis-price-letters-to-the-editor-1409346750).

For sure, some big pharmaceutical companies will be able to keep their R&D productivity high by investing in breakthrough innovation. The overall industry however, may not be able to compensate the reduced R&D efficiency solely by launching commercially high value (blockbuster) drugs. In contrast, the extraordinarily high R&D costs will make it for some pharmaceutical companies increasingly difficult to meet the investors’ expectations for a reasonable ROI exclusively from new products.

## The low R&D efficiency necessitates far reaching consequences for research-based pharmaceutical companies

**Fig. 2:**
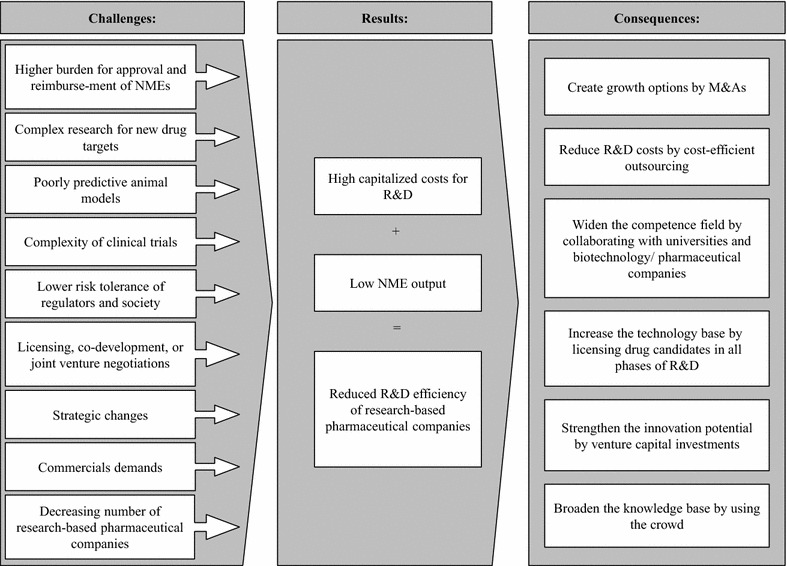
Challenges and consequences of the low R&D efficiency. *NME* new molecular entity, *M&A* merger and acquisition, *R&D* research and development

In the last years, more and more pharmaceutical companies realized that their low R&D efficiencies necessitate changes to their R&D ecosystems. In an analysis of major research-based pharmaceutical companies it was shown that 73 % of the investigated companies were making process changes in R&D [36], such as by (see Fig. 2):

- Creating growth options with M&As,
- Improving R&D efficiency by restructuring R&D into better manageable smaller and biotechnology-like units [37, 38],
- Reducing R&D costs by benefiting from virtual R&D and increasingly using cost-efficient outsourcing,
- Widening the competence field by progressively expanding collaborations and research partnerships,
- Increasing the technology base by more and more accessing drug candidates in all phases from external sources,
- Strengthening the innovation potential by venture capital investments, and
- Broadening the knowledge base by using the crowd.

Today, all major research-based pharmaceutical companies use opportunities along the whole R&D value chain to access external innovation. While Novartis (https://external-novartis.idea-point.com/Default.aspx), Sanofi (http://en.sanofi.com/partners/being_our_partner/being_our_partner.aspx) and AstraZeneca (https://www.astrazeneca.com/our-science/partnering.html) use the potential of more traditional collaboration and partnering types (including corporate venture capital funds), other pharmaceutical companies have set-up alternative open innovation models ranging from innovation centers, crowdsourcing, open source innovation to virtual R&D.

## Research collaborations

Traditionally, the pharmaceutical industry has collaborated with third parties to access specialty know-how [39]. As the complexity of pharmaceutical R&D increased fundamentally, nowadays collaborations are more and more used to get access to the required enlarged set of skills and technologies, such as novel drug targets, validation of targets, signal transduction pathway know-how, animal models, disease expertise, translational medicine know-how and biomarkers. As for example, GSK is spending nearly half of its R&D budgets to collaboration partners from academia or the biotechnology industry [40]. In this context, it has established Discovery Partnerships with Academia (DPAc), an alliance program starting from early screening to late optimization in any disease area and any treatment modality. While the academic collaboration partner can profit from GSK’s know-how in drug discovery and from its resources in medicinal chemistry, preclinical safety and pharmacokinetics, GSK can access ideas from academia to boost its innovation potential (http://www.dpac.gsk.com). In some areas of research, pharmaceutical companies have even broke new grounds by moving their proprietary technologies, such as HTS, to a larger number of external academic and institutional laboratories to increase flexibility and to benefit from governmental funding [41]. According to Frye [42], 78 academic screening centers focusing on high-risk drug targets were started in the USA till 2010. In Germany, the Lead Discovery Center (LDC) has been established in 2008 by the technology transfer organization Max-Planck-Innovation (MPI). The LDC aims at building a translational bridge between the excellence and know-how in basic research of Max-Planck scientists and applied pharmaceutical research, as for example by offering HTS technologies (http://www.lead-discovery.de/en/). Merck & Co. have started to launch in-depth academic partnerships with universities and academic institutes, such s the California Institute for Biomedical Research (Calibr), to translate academic basic research into new drugs (http://www.merck.com/licensing/partnership_success/academic_partnerships.html). Next, numerous pharmaceutical companies have closed their traditional R&D sites and opened new ones in close location with world-class academic institutions to better profit from their excellences and competences. As for example, Pfizer opened a new research site in Cambridge, Massachusetts, in 2014, after closing several sites following the merger with Wyeth (http://www.pfizer.com/research/science_and_technology/rd_locations/ma_cambridge). The new facility brings together around 1000 employees from Pfizer in one of the most well-known science hubs with famous universities, such as the Massachusetts Institute of Technology (MIT) or Harvard University, and more than 150 companies of the biopharmaceutical/biotechnology sector, amongst others Eli Lilly, Millennium (Takeda), AstraZeneca, Sanofi, GSK and Novartis (https://data.cambridgema.gov/Planning/Life-Sciences-and-Technology-Listing/fv53-bvhy). Also the academic partners profit from the close collaborations with pharmaceutical companies, as for example the Havard’s Office of Technology Development estimated that 4–5 % of its funding comes from industry collaborations [43]. In sum, collaborations (in different forms) between pharmaceutical companies and academic institutions/biotechnology companies are the norm today, building complex collaboration networks with pharmaceutical companies being the nodes of the networks [42, 44].

## M&As and project acquisitions

Since the financial crisis of 2007, M&As have become increasingly important in the biopharmaceutical sector. Pharmaceutical companies use M&As to compensate revenue losses of blockbuster patent expirations, to access strategically important intellectual property (IP), to exploit technology-based treatment innovations, to develop new core competencies, or to fill R&D pipeline gaps.

Most of the research-based pharmaceutical companies widened the breadth of their portfolio by accessing research projects and drug candidates from external sources to supplement their in-house pipeline and to meet at least part of their growth objectives by product innovation. Today, 50 % of the R&D pipelines of multinational pharmaceutical companies come from external sources [44].

## Portfolio management

Another approach to increase R&D efficiency is a greater focus on portfolio management and, thus, on project-related costs and project ROI. For example, DiMasi et al. reported a decrease in the average time from start of a research project to its abandonment in clinical trials by 30 % from 4.7 years to 3.3 years, indicating a trend towards earlier decision-making which reduces R&D costs as drug candidates fail earlier and cheaper [35].

According to the principles of modern value-based portfolio management, a portfolio of projects must be large enough to compensate project failures in drug discovery and development. While individual projects fail because of technical, commercial or market risks, the rest of the drug project portfolio must to be robust enough to provide the ROI expected by investors. The bigger the project portfolio the easier drug project failures can be compensated. As a consequence, the individual pipeline size of pharmaceutical companies increased in the past years [45]. Today, the corporate R&D pipelines of the top companies include more than 150 drug projects in development phases, with GSK (261), Roche (248), Novartis (223), and Pfizer (205) having 200 and more drug projects in their portfolio [45].

## R&D cost cuts

Some pharmaceutical companies analyzed the saving potential of R&D and cut their units to increase their R&D efficiencies. Principally, a reduction in R&D costs is combined with a release of R&D personnel and outsourcing of R&D activities to service providers in low-cost countries to reduce operational and infrastructure costs. GSK announced in 2012 to realize annual savings of GBP 1 billion by 2016 by reducing the size of its R&D and manufacturing organizations (http://www.pharmatimes.com/article/13-02-07/GSK_puts_faith_in_pipeline_and_cuts_costs_after_tough_2012.aspx). Merck & Co. published to reduce its R&D, manufacturing and administration staff by 8.500 people which should result in a USD 1.25 billion cost saving (http://www.fiercepharma.com/story/skinny-earnings-cost-cuts-boost-merck-bristol-myers-forest-fx-hits-sanofi/2014-04-29). Takeda’s new CEO, Christophe Weber, aims to reduce the R&D costs by specialization and focus on therapeutic areas where Takeda has a leading position (http://www.fiercebiotech.com/story/takeda-preps-stringent-rd-new-boss-takes-reins/2014-08-05). The change is in line with its USD 1 billion cost cutting program (http://www.fiercepharma.com/story/takedas-new-outsider-cfo-charged-1b-cost-cutting-plan/2013-11-18). Pfizer has been very active in reducing its costs after executing two major mergers since 2003, when more than 50,000 employees lost theirs jobs (http://www.fiercepharma.com/story/pfizers-post-megamerger-cost-cutting-record-51500-jobs-7-years/2014-04-29) and annual R&D expenditures were reduced from USD 9.4 billion (2010) to USD 6.7 billion (2013).

## Outsourcing

Today, outsourcing and collaborations with service providers are a standard in the pharmaceutical sector. Outsourcing companies are providing services along the whole value chain from research to development, marketing and manufacturing [46–48]. The global drug discovery outsourcing market was USD 14.9 billion (2014) and is expected to reach USD 25 billion by 2018 [49], while the market for CRO-conducted clinical trials was USD 23.1 billion (2014) and is expected to increase to USD 35.8 billion by 2020 (http://www.pharmsource.com/market/how-big-is-the-market-for/). What has been outsourcing on demand with many external service providers and redundant internal functions will become an integrated model of outsourcing with a limited number of strategic partners and long-term relationships [50].

## Innovation centers

The industry has also realized that innovation centers may be a driving force for creativity and innovation, a smart way to bring together company-internal and external experts and to integrate internal and external know-how to solve R&D challenges. As for example, GSK launched in 2007 its Center of Excellence for External Drug Discovery (CEEDD), an externally focused R&D center that facilitates drug discovery alliances up to clinical proof-of-concept (PoC) with external partners working across all therapeutic areas (http://www.out-sourcing-pharma.com/Preclinical-Research/GSK-opens-Centre-of-Excellence). It combines the model of a biotech alliance with the principles of a virtual organization and it provides diversity through externalization. CEEDD’s partners bring in their technologies and drug compounds while GSK is providing drug discovery and development expertise and services. If the drug candidate is showing proof-of-concept in clinical development, GSK is seeking a worldwide license for full development and commercialization. GSK’s external drug discovery activities are supported by the in-house scientific consultancy Scinovo that manages the interface of external partners and internal scientists (http://www.gsk.com/en-gb/business-to-business/discovery-and-development-consulting/). Till today, CEEDD helped to fill GSK’s early stage pipeline that consists now to 50 % of externally sourced projects (http://www.glaxosmithkline.at/common/pdf/130311_GSK-auf-der-Life-Science-Success_2013.pdf).

Pfizer established the Global Centers for Therapeutic Innovation (CTIs) in 2010, an open innovation model that aims at founding global partnerships between Pfizer and academic medical centers (http://www.pfizer.com/research/rd_works/centers_for_therapeutic_in-novation.jsp). While Pfizer is providing financial funding, human resources and technologies, the academic partners bring in their hypotheses of new drug mechanisms. Decision-making is done in joint steering committees. The inventions are filed in the name of both partners with Pfizer having the right of first refusal [51].

J&J is also investing in improving its network to entrepreneurs, startup companies, researchers, academic institutions and external innovators. To support its effort, J&J has even set up six dedicated sites, such as in San Diego or at the Texas Medical Center (http://jlabs.jnjinnovation.com). Thereby, the company aims to support early research projects in fields of oncology, immunology, neuroscience, cardiovascular and metabolism, infectious diseases and vaccines to provide the collaborators the necessary technical and financial resources to bring a product to marketability.

## Open source

The open source philosophy is based on transparency, freedom-to-operate, access to results and products for everybody, collaborative improvements, no financial reward for contributors, but recognition in providing a better solution to a challenge. Although these principles do not fit in the context of the IP-driven pharmaceutical sector, some pharmaceutical companies entered the arena of open source innovation. For example, GSK together with Alnylam Pharmaceuticals and the MIT have formed the Pool for Open Innovation against neglected tropical diseases (NTDs) providing open access to 2300 patents in respect to the treatment of tropical diseases (http://investors.alnylam.com/releasedetail.cfm?ReleaseID=466757). GSK also collaborates with Bayer and Novartis in the Global TB Alliance (http://partnerships.ifpma.org/partnership/global-alliance-for-tb-drug-development-tb-alliance). Although GSK’s has been focusing on neglected diseases so far, it also started to apply this open innovation model to other therapeutic areas, such as to infectious and rare diseases or to its clinical trial data [52]. Other examples of open source models in the pharmaceutical industry are the Open Source Drug Discovery initiative (http://www.osdd.net/home) that aims at providing affordable healthcare for neglected diseases and the African Network for Drugs and Diagnostics Innovation (ANDI) that was launched in 2008 (http://www.andi-africa.org) [53–55].

## Crowdsourcing

Eli Lilly is a pioneer and leader in the crowdsourcing field in the pharmaceutical industry. It initiated several crowdsourcing initiatives such as Innocentive^®^ or YourEncore- both are now operated independently. YourEncore (www.yourencore.com) is an expert network working in technology industry, such as life science, consumer and food industries, that support companies to access expert know-how to help to solve the companies’ problems. Fields of expertise in the pharmaceutical industry are preclinical and clinical development, clinical operations, manufacturing, regulatory affairs, organizational effectiveness, safety, pharmacovigilance, and quality management. Innocentive^®^ (www.innocentive.com) is a global network of more than 365,000 registered problem solvers coming from about 200 countries and problem-posting companies, such as AstraZeneca, Eli Lilly, NASA, Procter & Gamble, Syngenta, have partnered with InnoCentive^®^ to get innovative ideas provided. More than 2000 external challenges and more than 40,000 solutions were posted since the start of Innocentive^®^ in 2001, and more than 1500 rewards have been given so far.

Alternatively, the crowd can bring in new ideas, such as target proposals, that are sourced to the R&D pipeline if evaluated positively. In 2009, Bayer Healthcare has started its crowd-sourcing platform Grants4Targets where it offers two types of grants of EUR 5000–10,000 and EUR 10,000–125,000 for anyone who, for example, submits a target structure that is interesting for research [56]. The crowdsourcing platform receives noticeable global recognition, as around 2000 interested experts click the website per month. So far, most of the proposals came from Germany (21 %), Europe (except Germany, 39 %) and the US (23 %) in the fields of oncology (64 %), cardiology (26 %) and gynecology (8 %). Most of the target approaches were small molecules (63 %). Until today, more than 1110 applications were filed, 13 % of which were accepted and rewarded with a total sum of EUR 3.2 million resulting in 6 lead generation, one lead optimization and two preclinical development projects [57].

## Virtual R&D

A virtual R&D model can be defined as an organization which works with a limited number of internal staff and which uses external resources, technologies and facilities on demand to develop their R&D projects efficiently. Although a virtual R&D model provides numerous advantages, such as reduced capital requirements and financial risks, reduced overhead costs, limited infrastructure costs, or higher flexibility, the model has so far only successfully applied by Chorus, Shire, Protodigm, Debiopharm, and Endo Pharmaceuticals.

Chorus, an entity of Eli Lilly, has proven that virtual R&D can help to reduce both cost and time needed in pharmaceutical R&D (www.choruspharma.com). In 2002, Chorus started as an alternative path for drug R&D with the aim to manage the complex R&D process lean and flexible and to bring preclinical compounds with earlier decision in a “lean-to-PoC” model to the clinical PoC in a shorter time and at lower costs. Chorus manages a portfolio of 15–17 projects in the phases of candidate selection to PoC in 19 countries with around 40 full-time employees in a flat hierarchy model—all experts in the Chorus team report to one managing director. Approximately 25 % of Chorus’ budget are fixed overhead costs, the remaining 75 % are allocated to the external costs of the drug projects [58]. The success of Chorus is outstanding, as since 2002, the productivity of Chorus has been 3–10 times higher than the traditional pharmaceutical R&D model of Eli Lilly—in particular the improved PTRS in Phase II provided the greatest potential to increase the R&D efficiency [59].

Shire may have established the most radical concept in the pharmaceutical sector so far, as the whole R&D organization operates virtual as a knowledge leverager. As presented in a previous report, the top innovators of the industry usually follow the knowledge creator or the knowledge integrator models [44]. The knowledge creator is an open innovator type whereby the company has an inbound preference in innovation management combined with a preferentially internal generated project portfolio. And the knowledge integrator is a preference toward external generated R&D projects in combination with in-house expertise in R&D management. Shire has drafted its new innovation model that combines several open innovation aspects into one coherent concept that helps to increase R&D efficiency [44]. Shire has a trim R&D team that is almost a virtual network with low overhead costs. It focuses on external generated innovation in combination with a predominantly outside-oriented way of innovation management. It acquires ideas, know-how and technologies from other companies and universities to discover and develop new drugs that come primarily from external sources. This model offers the possibility to reduce attrition by selecting the right portfolio of low-risk projects. It also provides the opportunity to manage the project pipeline effectively by accessing projects and resources from the outside flexibly. And it allows the option to access resources cost efficiently, as resources can be accessed globally with low overhead costs.

## Conclusions

The reduced R&D efficiency makes it necessary for pharmaceutical companies to realign their R&D concepts. Companies which aim to be the top innovators in the pharmaceutical industry can follow the knowledge creator or knowledge integrator models. In these models innovation is preferentially created internally or by integrating external assets. These companies need to identify the right growth strategies, need to build up the right core competences for drug R&D internally, need to build external networks with academic partners and service providers and, in particular, need to accept the high costs for product innovation and ensure a sustainable investment in R&D to generate a steady flow of new innovative drugs.

Pharmaceutical companies, which are not counted to be a top innovator, may still be successful when focusing on the growth options that are provided by the generics business and the emerging markets. While the worldwide drug prescription market is forecasted to grow at around 6 % annually between 2015 and 2020, the generics business will grow by 12 % per year in the same time [1]. And, already by 2016, it is expected that the pharmaceutical industry is generating around one third of its total sales in the emerging markets [60]. The financial potential in these countries is forecasted to be USD 500 billion by 2020 [61]. As a consequence, some of the multinational pharmaceutical companies have changed their business models from purely research-based pharmaceutical companies that focused on the traditional pharmaceutical markets to more diversified companies and are already generating today a major part of their total revenues outside of Europe, US and Japan by selling both innovative medicine and generic drugs [1].

Finally, research-based pharmaceutical companies that cannot afford to diversify or to follow the knowledge creator and integrator models need to have an eye on other R&D concepts that are more appropriate for their set-up. Certainly, open innovation has proven to be a concept of significant attention for the pharmaceutical industry. Either it can be used to complement the traditional R&D model to increase the reach of the internal R&D organization, to access external innovation more easily and to reduce R&D costs. Research alliance concepts such as the CTI and crowdsourcing can be ranked as most valuable examples to improve the R&D efficiencies. Or, and applicable for organizations that are more open to a fundamental changes in their R&D models, it is recommended to follow the strategy of the knowledge leverager Shire which has demonstrated that this R&D concept can be translated into enhanced performance [44]. To become a knowledge leverager, pharmaceutical companies need to make the following modifications:

- increase their absorptive capacities by implementing open innovation processes,
- hire people who are open-minded, able to work with different cultures and aware that innovation need to be accessed globally,
- improve their dynamic capabilities and interpersonal skills,
- form more strategic alliances and active involvements in innovation networks, and
- develop managerial abilities to better utilize external partnerships.

## Footnotes

1 This review details the efficiency parameters of pharmaceutical R&D and the consequences of the low R&D efficiency for the industry. See also Schuhmacher et al. [10].
